# Improving Hydrolysis Characteristics of Xylanases by Site-Directed Mutagenesis in Binding-Site Subsites from Streptomyces L10608

**DOI:** 10.3390/ijms19030834

**Published:** 2018-03-13

**Authors:** Ke Xiong, Suyue Xiong, Siyu Gao, Qin Li, Baoguo Sun, Xiuting Li

**Affiliations:** 1 Beijing Innovation Centre of Food Nutrition and Human, Beijing Technology & Business University (BTBU), No. 33 Fucheng Road, Haidian, Beijing 100048, China; xiongke@btbu.edu.cn (K.X.); xiongsuyue2017@foxmail.com (S.X.); gaosiyu_smile1@163.com (S.G.); 17888820100@163.com (Q.L.); lilite1948@aliyun.com (B.S.); 2 Beijing Laboratory for Food Quality and Safety, Beijing Technology & Business University (BTBU), No. 33 Fucheng Road, Haidian, Beijing 100048, China; 3 Beijing Engineering and Technology Research Center of Food Additives, Beijing Technology & Business University (BTBU), No. 33 Fucheng Road, Haidian, Beijing 100048, China; 4 Beijing Key Laboratory of Flavor Chemistry, Beijing Technology and Business University (BTBU), No. 33 Fucheng Road, Haidian, Beijing 100048, China

**Keywords:** GH10/11-xylanase, binding site, Xylooligosaccharide

## Abstract

The preparation of oligosaccharides via xylan hydrolysis is an effective way to add value to hemicellulosic material of agricultural waste. The bacterial strain Streptomyces L10608, isolated from soil, contains genes encoding xylanases of glucoside hydrolase family 10/11 (GH10/11), and these have been cloned to catalyze the production of xylooligosaccharide (XOS). To improve the XOS proportion of hydrolysates produced by xylanase, four amino acid residues were substituted by site-directed mutagenesis, and the mutant genes were overexpressed in *Escherichia coli*. Mutations replaced the codons encoding Asn214 (+2) and Asn86 (−2) by Ala and removed the Ricin B-lectin domain in GH10-xyn, and mutants Y115A (−2) and Y123A (−2) were produced for GH11-xyn. Interestingly, GH10-N86Q had significantly increased hydrolysis of XOS and almost eliminated xylose (X1) to <2.5%, indicating that the −2 binding site of GH10-xyn of L10608 is required for binding with xylotriose (X3). The hydrolytic activity of GH10-N86Q was increased approximately 1.25-fold using beechwood xylan as a substrate and had high affinity for the substrate with a low *K_m_* of about 1.85 mg·mL^−1^. Otherwise, there were no significant differences in enzymatic properties between GH10-N86Q and GH10-xyn. These mutants offer great potential for modification of xylanase with desired XOS hydrolysis.

## 1. Introduction

Xylooligosaccharides (XOS) are functional glycopolymers formed by 2–7 xylose (X1) molecules with glycosidic bonds, of which xylobiose (X2) and xylotriose (X3) are the main effective components [1]. Preparation of oligosaccharides via xylan hydrolysis is an effective way to add value to hemicellulose materials from agricultural waste. XOS has a wide array of biotechnological and industrial applications [2]. However, there are many problems in enzymatic production of XOS leading to difficulties in industrial applications [3], such as the high cost of purification and low proportions of effective components. At present, when xylanase from microorganism hydrolyzing xylan, the content of specific effective components in production, such as xylobiose (X2) and xylotriose (X3) are mostly less than 30%. The production also contains a large number of non-effective monosaccharide components, such as xylose and Arabia. A high cost of purification is required to increase proportions of effective components (60–70%) [4]. Solving these problems depends on screening for natural xylanases that possess desired characteristics. However, like many xylanases [5,6,7], only a few xylanases possess special hydrolysis characteristics. The ratio of X2 and X3 were more than 50% in their hydrolysis products [8,9,10]. Each xylanase has differences in its hydrolysis activity, and it is a challenge to catalyze high yields of XOS while eliminating or reducing the production of X1 and avoiding further hydrolysis of XOS.

Extensive research has shown that the mechanism of xylanase is acid-base catalysis [11]. Most active sites in xylanases are two Glu residues: one as an acid/base and the other as a nucleophile. Besides the active sites, xylanases display some binding subsites, which are defined as contiguous regions of the enzyme constituted by residues that interact in a non-covalent fashion with monomeric units of the substrate [12,13]. Binding sites of xylanases are usually located in an open cleft of the catalytic domain, which are defined as −n and +n; the hydrolysis reaction occurs between −1 and +1 sites [14]. Factors that affect hydrolysate production include differing the raw material and preparation of xylan, the degree of polymerization (DP), water-solubility, the sugar base ratio, branched chain groups, and residual lignins and salts [15,16]. The amino acid sequence and structure of xylanase in particular will affect the yield of XOS [17,18]. Substrates need to bind with the catalytic domain in the enzyme, and substrates with different DP and spatial structures that affect stable binding will have a great effect on product formation [19,20].

The bonds and catalytic transition state formed by different binding sites in the enzyme are also diverse. Xylanase from different families may require substrates to bind with different binding sites to generate XOS products with different DP. Vandermarliere et al. [21] indicated that differences between hydrolysis products are due to the aglycone subsites or to subtle changes at the glycone subsites. Binding sites of amino acid residues through hydrogen bonding and hydrophobic interactions are due to a binding of substrate and recognition sites [22,23,24], so different bindings can lead to corresponding differences in species and hydrolysate composition.

In addition to high XOS production from the wild-type xylanase, mutations K189A and N182A in xylanase WtXYLA from *Pseudomonas fluorescens* [25] increase the X1 and X3 ratio and decrease X2, while the N44A mutant increases the X2 proportion by about 20%. Yang et al. [26] studied the GH11 xylanase rAfxynA of *Aspergillus fumigatus*, which produces mainly X2, but site-directed mutagenesis of three amino acid residues (N70A, R121A, and Q135A) led to the amounts of X1 and X3 to rise while X2 decreased. BsxynA from *Bacillus subtilis* [27] has Tyr88 at the +3 binding site, which has an important influence on substrate specificity; Y88A results in an increase in X1 and decrease in X3. The mode of action of endo-β-1,4-xylanases from families 10 and 11 are different from acidic xylanases, because branched chain methyl-glucuronic acid (MeGlcA) can be accommodated at position +2 of GH11 and +1 of GH10 endo-β-1,4-xylanases [28].

Although the amino acid residues involved in the formation of enzymatic hydrolysis products have not yet been fully identified, it is clear that amino acid residues play a decisive role in substrate selectivity and specificity of hydrolysis products of xylanase. Some constructed mutants have increased the proportions of effective components such xylotriose and xylobiose, but the amounts of xylose non-effective components were also higher in previous studies. Most of THE mutant sites in xylanases are close to the catalytic site, which have great influence on the enzyme activity in these constructed mutants. The present study focused on the substrate binding site, which is related to product specificity, to explore the binding mode of the enzyme. The enzyme combined with the substrate via non-covalent interaction (such as hydrogen bonds and hydrophobic interactions) between the amino acid residues in xylanase and the substrate. The amino acid residues with different types and locations in the catalytic domain of xylanase led to different binding modes between enzymes and their substrates. Binding sites ±1 and ±2 in xylanases are close to the catalytic site, which have great influence on the binding mode between xylanases and their XOS substrates. Based on these key binding sites, we constructed mutants to elucidate the mechanism. These constructed mutants possessing special hydrolysis characteristics will reduce the high cost of purification and improve the proportions of effective components in enzymatic production, which provides a feasible way for enzymatic methods to produce XOS in industrial applications.

## 2. Results

### 2.1. Isolation and Identification of the Xylanase from Streptomyces L10608

**Figure 1:**
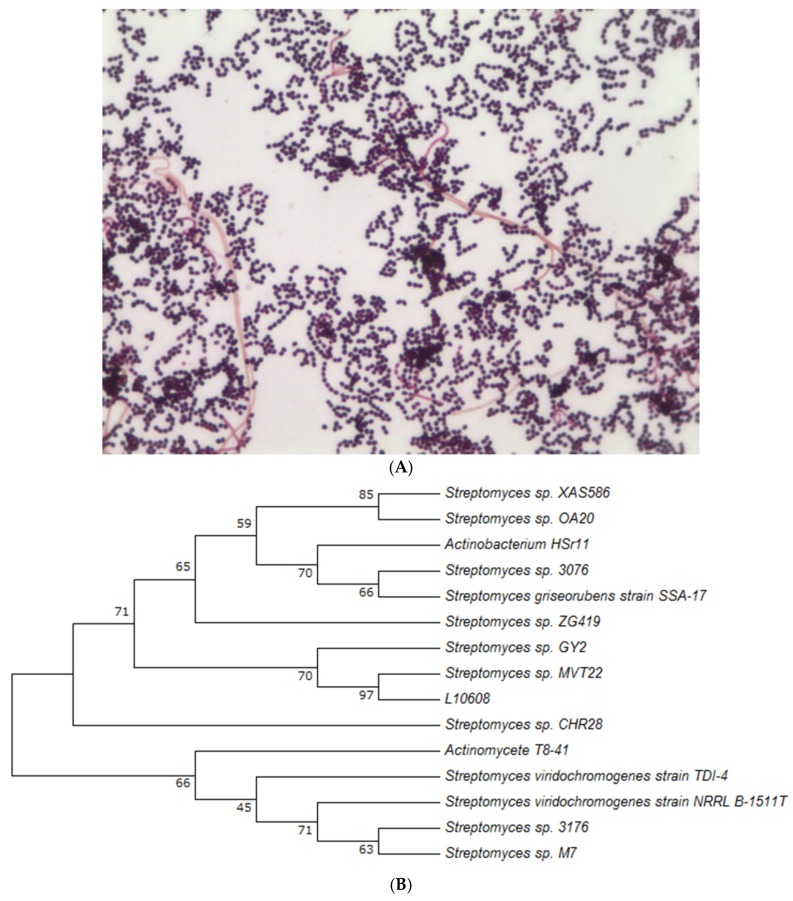
The morphological characteristics of strain L10608 on a microscope (1000×) (**A**) and phylogenetic tree of L10608 (**B**).

Strain L10608 was identified by 16S rRNA through alignment and cladistics analysis of known homologous nucleotide sequences, phylogenetic relationships were inferred, and the approximate phylogenetic position of the strain was determined (Figure 1).

**Figure 2:**
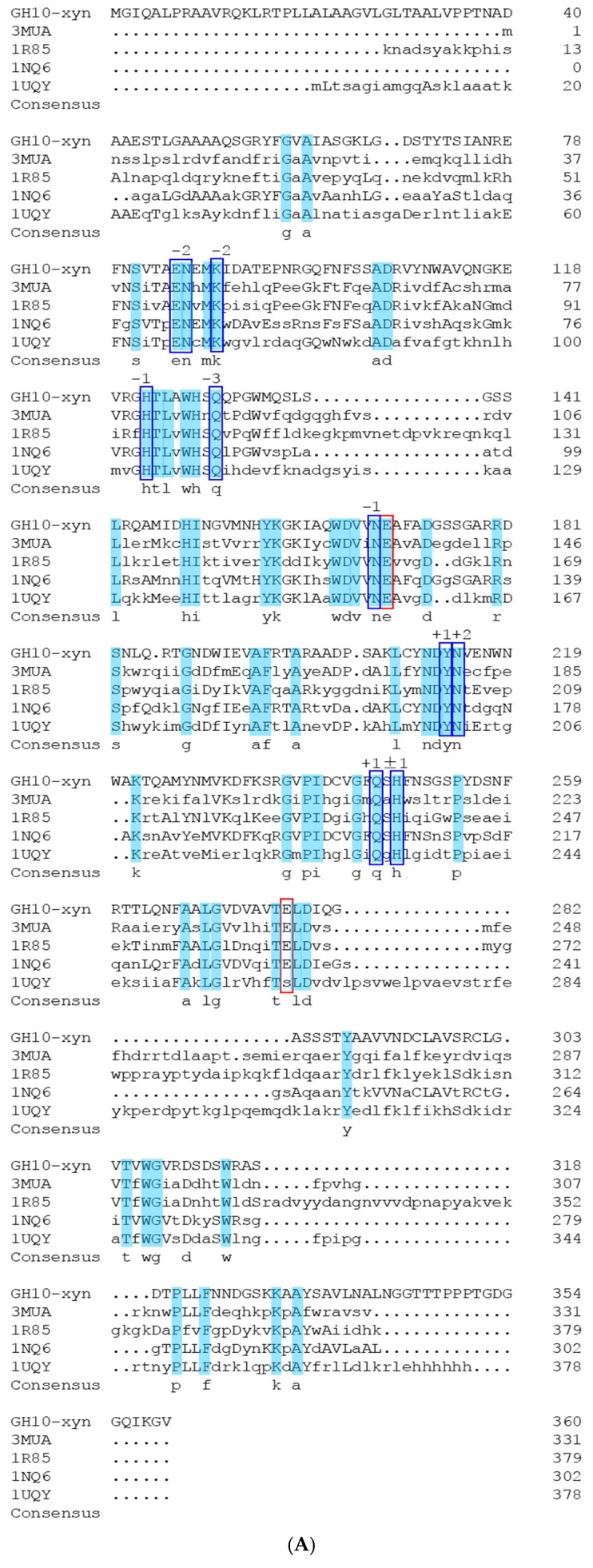

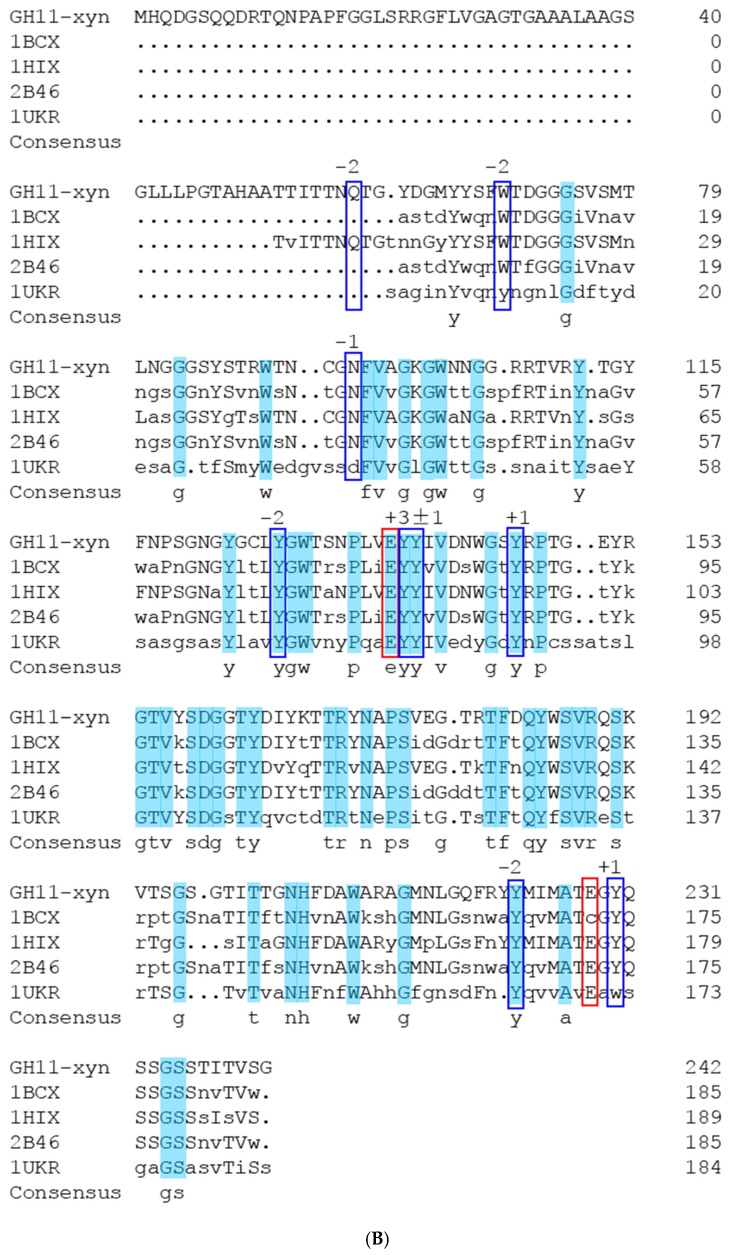
The sequence homology analysis of GH10-xyn (**A**); GH11-xyn (**B**) and others xylanases that has high similarity. Sequences be named according to their Protein Data Bank (PDB) code, which have identified the binding sites by crystallographic study. The grey color scheme indicated the conservation of amino acids. The red and blue squares are the catalytic sites and binding sites, respectively.

The *GH11-xyn* sequence of the *Streptomyces* L10608 was searched in NCBI and found to be identical to that of 1,4-β-xylanase (*Streptomyces* sp. JHA19) (NCBI accession number: WP_055619964), and the GH10 xylanase from *Streptomyces* L10608 was identical to that of the same strain as GH11 (*Streptomyces* sp. JHA19) (GenBank accession number: WP_055619031). Both sequences are 100% similar with *Streptomyces* sp. JHA19. (Figure 2).

The bioinformatics analysis predicted that *GH10-xyn* gene has a total length of 1431 bp (Figure S1), while molecular weight of the protein is 50.677 kDa (the theoretical isoelectric point: 6.98). The signal peptide of the *GH10-xyn* gene is MGIQALPRAAVRQKLRTPLPALAAGVLGLTAALVPPTNADA. The base composition ratio in the *GH10-xyn* gene is 18.2% A, 38.4% C, 30.5% G and 12.9%T (The base composition ratio in *GH10-xyn* gene was analyzed by the online analysis software, http://web.expasy.org/protparam/). Its protein sequence has a homologous region in the glucoside hydrolase family 10 (GH10) and encodes a Ricin B-lectin domain consisting of 108 amino acids. The *GH11-xyn* gene has a total length of 729 bp (Figure S1), while molecular weight of protein is 22.446 kDa (theoretical pI isoelectric point: 8.98). The signal peptide of *GH11-xyn* gene was MHQDGSQQDRTQNPAPFGGLSRRGFLVGAGTGAAALAAGS. The base composition ratio of *GH11-xyn* gene is 19.5% A, 36.6% C, 30.6% G and 13.3% T (The base composition ratio in *GH11-xyn* gene was also analyzed by the online analysis software, http://web.expasy.org/protparam/). Its protein sequence has a region that is homologous in the glucoside hydrolase family 11 (GH11).

### 2.2. Structural Analysis and Site-Directed Mutagenesis of Streptomyces L10608

DNA alignmentAnalogous 3D structure analysis showed that the catalytic center of GH11-xyn was located in a cleft formed in the middle of the overall-sandwich structure, and GH10-xyn had a smaller binding region [24].

**Figure 3:**
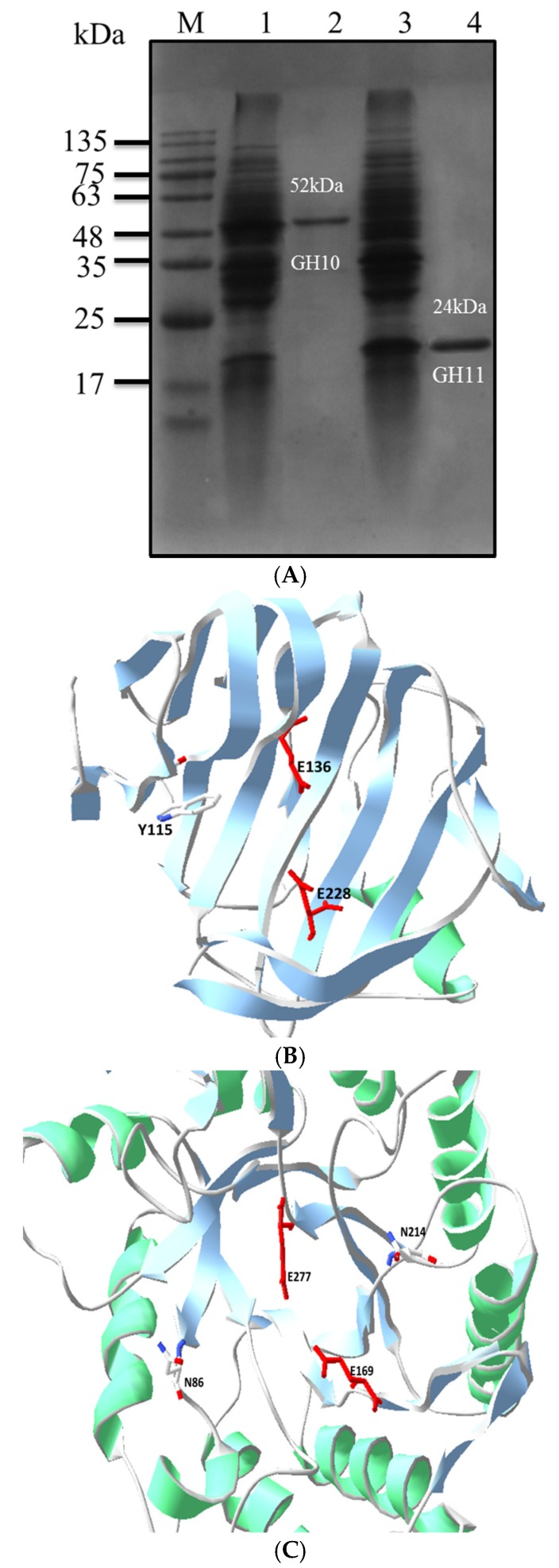
The SDS and Modeling locations of the enzyme. (**A**) The SDS of crude and purified enzyme. M: Mark, Channel 1: crude enzyme of GH10-xyn, Channel 2: purified enzyme of GH10-xyn, Channel 3: crude enzyme of GH11-xyn, Channel 4: purified enzyme of GH11-xyn. (**B**) Modeling locations of the binding sites and catalytic sites of GH10-xyn and (**C**) Modeling locations of the binding sites and catalytic sites of GH11-xyn. Red amino acid is the catalytic sites, white amino acid is binding sites. Y115 is −2 binding site of GH11-xyn and N86, N214 are binding sites of GH10-xyn. Green structures are α-helix, blue structures are β-sheets.

Sodium dodecyl sulfate polyacrylamide gel electropheresis (SDS-PAGE) analysis indicated that the molecular weights of GH11-xyn and GH10-xyn proteins were about 24 and 52 kDa, which were identical to the predicted values and are characteristic of different glucoside hydrolase family members. To evaluate the enzyme characteristics, the wild-type and mutants were cloned into pET vector expressed in *E. coli* BL21 and were further purified using the Ni-sepharose (Figure 3A).

Based on the structure and sequence being highly similar within the catalytic domain [29,30], based on our 3D structure model (Figure 3B,C), potential binding sites of strain L10608 were determined by homologous alignment with known binding sites of highly similar xylanase sequences (Figure 2).

**Figure 4:**
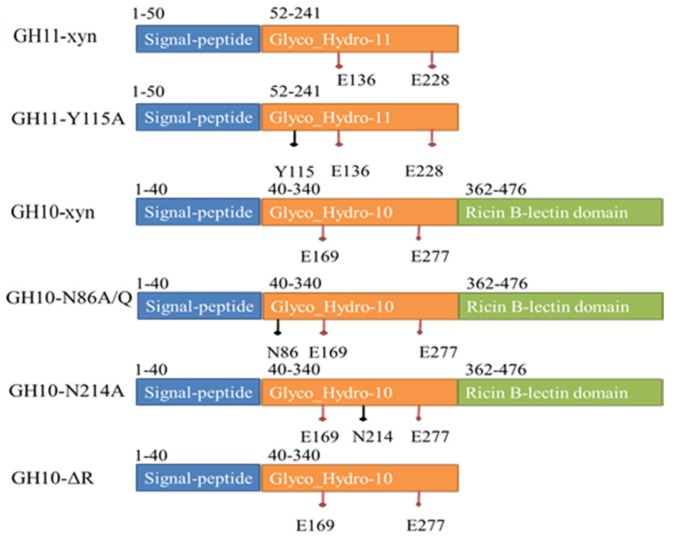
Schematic overviews of xylanases produced from Streptomyces L10608. GH11-xyn: GH11 family recombinant xylanase from Streptomyces L10608. GH11-Y115A: binding site on −2 site-directed mutagenesis of GH11 xylanase. GH10-xyn: GH10 family recombinant xylanase from Streptomyces L10608. GH10-N86A/Q: binding site on −2 site-directed mutagenesis of GH10 xylanase. GH10-N214A: binding site on +2 site-directed mutagenesis of GH10 xylanase. GH10-ΔR: Remove Ricin B-lectin domain of GH10 xylanase.

Site-directed mutagenesis and creation of *GH10-ΔR* was carried out by gene splicing and overlap extension PCR (SOEPCR) [31]. Schematic overviews of xylanases produced from Streptomyces L10608 ae shown below. When the xylanase combined with the xylan substrate binding sites of xylanases are usually located in an open cleft of the catalytic domain, the hydrolysis reaction occurs between −1 and +1 sites, which are very important to maintain the hydrolytic activity of xylanase. Thus, ±1 sites were too close to the catalytic site to modify. Based on the structure and sequence alignment, site-directed mutagenesis focused on the ±2 binding sites. GH10-xyn removed the Ricin B-letin domain which was also constructed to explore the hydrolysis effect of this structure. GH11-xyn stands for GH11 family recombinant xylanase from Streptomyces L10608. GH11-Y115A means binding site on −2 site-directed mutagenesis of GH11 xylanase. GH10-xyn stand for GH10 family recombinant xylanase from Streptomyces L10608. GH10-N86A/Q indicate the binding site on −2 site-directed mutagenesis of GH10 xylanase. GH10-N214A means binding site on +2 site-directed mutagenesis of GH10 xylanase. GH10-ΔR stands for removing Ricin B-lectin domain of GH10 xylanase. Site-directed mutant Y115 (−2 Binding site in GH11-Y115A) and mutant N214 (+2 Binding site in GH10-N214A) were located between two catalytic sites. Site-directed mutant N86 (−2 Binding site in GH10-N86A/Q) was located at the Nitrogen terminal from the catalytic site. The Ricin B-lectin domain removed in Mutant GH10-ΔR was located at the carbon terminal in GH10 xylanase (Figure 4).

### 2.3. Analysis of Kinetic Parameters and Enzymatic Characteristics

Kinetic parameters of all purified recombinants were examined (Table 1). The original activities of GH10-xyn and GH11-xyn were 160 U·mL^−1^ and U·mL^−1^, respectively. Among the mutants, GH10-N86Q had the consistent activity of GH10-xyn with a similar Michaelis constant. However, its *K_cat_* decreased to 94.90 s^−1^ from 150.82 s^−1^ of the wild-type. GH10-N86A and GH10-N86Q showed weakened −2 binding capability. It speculates that Ala (GH10-N86A) substituted for Asn at the −2 subsite weakened substrate binding, while the Gln (GH10-N86Q) substitution retained the activity as predicted by molecular structure modeling. Thus, GH10-N86Q had a consistent activity of GH10-xyn. *K_cat_* refers to the reaction constant (catalytic constant) and indicates the ability of the enzyme to catalyze specific substrates; the higher the *K_cat_* value, the higher the catalytic rate of the enzyme. The GH10-N86Q site-directed mutagenesis at the −2 site partially weakened the affinity of the substrate and enzyme with a 1.85 mg·mL^−1^ *K_m_* value. *K_m_*, commonly known as the Michaelis constant, can reflect the affinity between enzyme and substrate; the lower the *K_m_*, the greater the affinity. The *K_cat_*/*K_m_* of GH10-N86Q was the highest among the mutants at 51.29 mg^−1^·s^−1^ mL. *K_cat_*/*K_m_* reflects the affinity and catalytic ability of enzyme towards a substrate and is used to measure the enzyme catalytic efficiency of different substrates.

GH10-N86A and GH11-Y115A mutants with mutations at the −2 subsites showed relative xylanase activities that were significantly decreased to 8.28% and 6.25%, respectively. GH10-N86A tended to have a higher Michaelis constant (13.01 mg·mL^−1^) and much lower *K_cat_* (10.68 s^−1^) than the wild-type (1.64 mg·mL^−1^ and 150.82 s^−1^), in accordance with its low activity and *K_cat_/K_m_* (0.82 mg^−1^·s^−1^ mL). Substitution of Asn214 for Ala in the +2 subsite decreased activity to 74.15% of the wild-type and had a negative effect on *K_m_*; *K_cat_* was reduced to about 1/3 of the original value, and *K_cat_/K_m_* was low at 17.56 mg^−1^·s^−1^ mL. Furthermore, GH10-ΔR had little impact on the Michaelis constant, while the *K_cat_* and *K_cat_/K_m_* were also strongly reduced to about of the original value.

**Figure 5:**
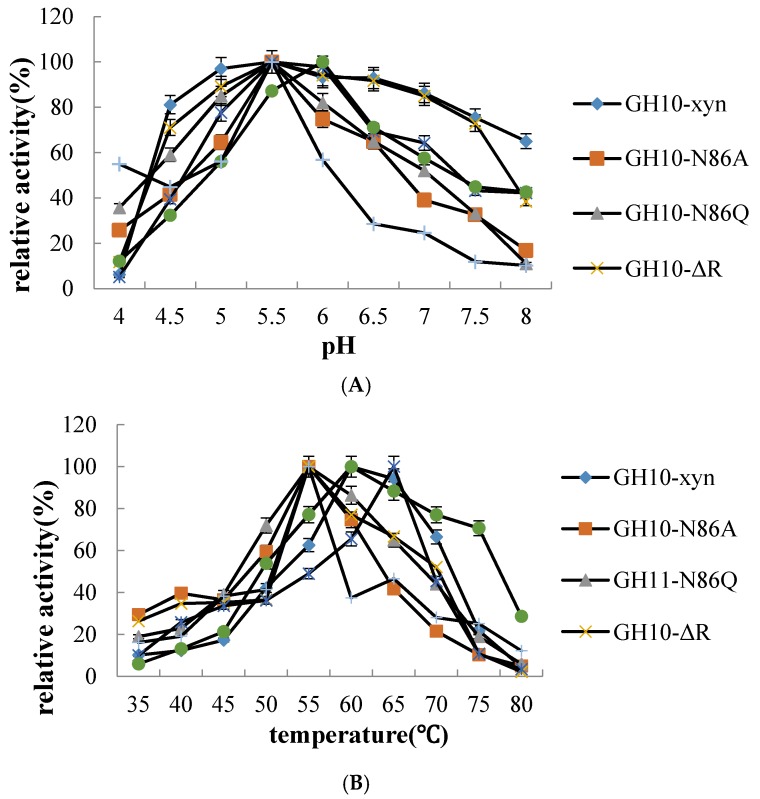
The properties of GH10/11-xyn and mutants with beechwood xylan substrate (**A**) optimal pH and (**B**) Optimal temperature. The best xylanase activity defined as 100%.

We examined the optimal temperature and pH (Figure 5). The optimal temperature of GH10/11-xyn wild-type was 60 °C, but for most mutants, it decreased to 55 °C, except for GH10-N214A which increased to 65 °C. GH11-Y115A, GH10-N86A/Q, and GH10-N214A showed a sharp decrease out of the range 50–60 °C. GH11-xyn had relatively high activity at high temperature and retained 70.67% activity at 75 °C, while all others were under 26% activity. The optimal pH of the xylanases was at 5.5 except for GH11-xyn at 6.0, which showed that all favored neutral and weak acidic environments of pH 5.5–6.0. Both GH10-xyn and GH10-ΔR had relatively high activities at high pH, which were 75.43% and 72.9% at pH 7.5, respectively, GH10-xyn retained 65.02% activity at 8.0 pH. GH11-Y115A had the best activity at very high pH with 10.23% at pH 8.9, while activity remained 54.95% at pH 4.0.

### 2.4. Analysis of Hydrolysis Products of Wild-Type

We found that GH11-xyn hydrolyzed xylan to X3 and X1 as the main components, while the hydrolysates of GH10-xyn were mostly X2 and X1. GH10-xyn catalyzed complete hydrolysis from X3 into 51.22% X1 and 48.78% X2, but GH11-xyn retained 36.72% X3. When GH10-xyn hydrolyzed X4, X2 reached 89.06% and rest was X1. The X1, X2, and X3 proportions of GH11 hydrolysates from X4 were 60.80%, 31.81%, and 7.39%, respectively; although X3 was still at a significant percentage, the lower yield may have been caused by X4 symmetrical hydrolysis into X2 [11]. The hydrolysate of X5 from GH10-xyn had no X3, and X2 was the highest accounting for 65.15%, while GH11-xyn hydrolysates contained X1, X2, X3, and X4 at proportions of 58.96%, 16.76%, 16.19%, and 8.09%, respectively. The GH11-xyn main hydrolysis products were X1 and X3, while for GH10-xyn, they were mostly X1 and X2. Interestingly, both xylanases had a strong capacity to hydrolyze one type of XOS over another (Table 2).

We also examined the hydrolysis of xylans to observe putative hydrolysis patterns for practical application. The results showed that the xylanases of strain L10608 have different hydrolysis abilities on beechwood xylan, oat-spelt xylan, and birchwood xylan (Table 3). The results of xylan hydrolysis were consistent with those of XOS, in which GH11-xyn prefers to hydrolyze X2 while GH10-xyn prefers X3. The accumulation of XOS by GH11-xyn and GH10-xyn was oat-spelt > beechwood > birchwood, and the hydrolysis yield of X2 was twice that for beechwood by GH10-xyn. The hydrolysis yields of X3 by GH11-xyn from oat-spelt xylan, beechwood, and birchwood were 2.05 mg·mL^−1^, 1.40 mg·mL^−1^, and 1.59 mg·mL^−1^, respectively. This is based in the analysis of hydrolysis products of mutants.

**Figure 6:**
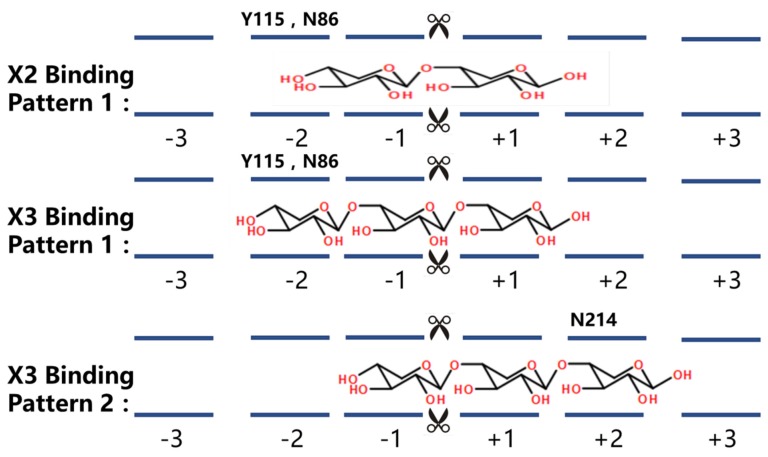
XOS and catalytic domain binding patterns.

We tried to improve XOS proportions according to hydrolysate characteristics and XOS binding patterns (Figure 6) by means of mutagenesis. Previous studies have demonstrated that substitutions of amino acids on the binding sites of some enzymes with alanine leads to a decrease in binding with the protein by disrupting non-covalent interactions [32]. Changing the enzyme sugar molecule binding mode leads to a change in the hydrolysate. We designed five xylanase mutants based on conservation of amino acid residues N214, N86, and Y115 in most GH10 and GH11 xylanases. From homologous alignment with existing crystal models, Y115 of GH11 and N86 of GH10 are in the −2 binding site, N214 of GH10 is in the +2 binding site, and both are very close to the predicted proton donor or nucleophile. Removal of the Ricin B-lectin domain that contains three disulfide bonds may not only change the structure of xylanase but also affect hydrolysates. Therefore, all the mutants were tested with DP 3–5 XOS to determine whether the hydrolysis mode changed (Table 2).

When the hydrolysates of GH10-ΔRicin and GH10-N214A were compared to the wild-type when X3 was the substrate, X1 decreased to 28.20% and 28.21% of the total hydrolysis product, respectively, but at the same time, X2 increased to 71.80% and 71.79%. When X4 was the substrate, X1 and X2 were slightly decreased, but still no X3 appeared. The hydrolysates from X5 had no X3, and X1 and X2 were slightly decreased while X4 disappeared.

In GH10-N86A, the changes in X3, X4, and X5 were desirable, although the yield of X2 decreased to not detected, 31.46%, and 28.87%, but that of X3 increased from 0% to 96.77%, 10.11%, and 52.92% of the total hydrolysis product, respectively, and most importantly, X1 no longer appeared. This indicated that the strong ability to hydrolyze X3 by GH10-xyn was weakened by the GH10-N86A substitution, and the binding site of −2 is vital to binding of X3 by xylanase. GH10-N86A successfully reduced X1 and enhanced the proportions of XOS on all substrates tested, but lost most of its activity, while GH10-N86Q not only achieved the same hydrolysate results (strong increase of X3 and near elimination of X1), but also increased activity by about 1.25-fold.

The effect of the GH11-Y115A mutation was not obvious. When using X3 as a substrate, GH11-Y115A, which is in the −2 binding site, significantly reduced X1 from 58.76% to 13.81% of the total hydrolysis product, which corresponds to the reserved X3 increase and the mostly unchanged X2. The mutation reduced the ability to hydrolyze X4, retaining 41.18% of it, while X1 and X2 were deceased to about 7% and X3 disappeared. It seems that the −2 binding site has a great impact on the binding of X4. Upon hydrolysis of X5, the proportion of X1 and X3 significantly decreased to 37.5% and 8.33%, while X2 and X4 increased to 25.00% and 29.17%, perhaps because the −2 binding site has more impact on X2 and X4 hydrolysis.

We also examined the hydrolysis of mutants using varying xylans for potential practical applications. Hydrolysates of GH10-xyn from the three xylan sources all contained 15–20% X1, and only retained 3.8% X3 from oat-spelt xylan hydrolysis, and most of the hydrolysate was X2. The GH10-N86Q mutant clearly improved the X3 proportions of hydrolysates using the three xylan sources as substrates, which demonstrates its value for practical application. When GH10-N86Q hydrolyzed the three xylans, X1 disappeared or was at only trace amounts under 2.5%, while X2 and X3 were at 27.78%, 17.8%, and 25.68%, and 53.76%, 46.53%, and 64.76% of the total hydrolysis product, respectively. X3 of GH10-N86A was also retained and replaced X2 to become the bulk of the hydrolysate. Consistent with the hydrolysis XOS, there was a very sharp increase in X3 and X4, which may also be the reason for the reduced X2.

GH10-ΔR and GH10-N214A showed similar changes in oat spelt and birchwood xylan hydrolysis, with X1 increasing to about 10% of the total hydrolysis product, while X2–X4 decreased. X_1_ decreased and X2 increased in hydrolysates of GH10-ΔR from beechwood xylan. GH11-Y115A unexpectedly decreased X1 to 0% and 4.0% when the substrate was beechwood or oat spelt xylan, respectively. X2 decreased as well, and X3 increased in both substrates to about 10%. X4 significantly increased to 10.12% and 26.95% from beechwood and oat spelt xylan, respectively.

## 3. Discussion

### 3.1. Role of the Ricin B-Lectin Domain Subsites in Substrate Binding and Hydrolysis by xynA

In this study, GH10-xyn had higher activity and more XOS production than GH11-xyn, while previously studies had indicated that GH10 xylanase noncatalytic domains, such as carbohydrate-binding domains or others, result in broad substrate specificity [33]. GH10-xyn shows strong hydrolysis of X3, while GH11-xyn does not. In many previous studies [16,34,35], GH10 xylanases have been shown to have higher activity against XOS and produce fewer products from glucuronoxylan and Araboxylan (AX) than GH11. Biochemical characterization of a GH10 xylanase has shown that it is highly versatile, releasing low DP XOS from high to low DP substrates and even xylose [36]. The high production of X2 may be due to the binding affinities of subsites −1 and +1 being negative, as xylobiose is a very unsuitable substrate for GH10 xylanases [22,37]. Normally, GH10 xylanase comprises not only a catalytic domain but also a carbohydrate-binding domain (CBD), which could lead to its broad substrate specificity [33]. The Ricin B-lectin domain belongs to the CBD 13 family, which can bind the polysaccharide xylan and retains the ability of the R-type lectins to bind low DP sugars such as lactose and galactose [38]. The domains of CBD 13 are able to bind mono- and di-saccharides that are too low in DP to involve the cooperation of second and third binding sites [39]. The diversity in CBD binding patterns may cause changes in GH10-ΔR with no obvious regularity.

### 3.2. Role of ±N Subsites of xynA in Hydrolysis

GH10-xyn and GH11-xyn showed improved XOS proportions by directed mutations of ±N subsites. The amino acids constituting the subsites interact with the substrate through hydrogen bonds or hydrophobic stacking interactions and have a crucial role in substrate recognition [40].

Previous research on *B. subtilis* xylanase [17] indicated that –O^2^ of Tyr69 forms a hydrogen bond with the –OH of xylose at the −2 site, and the same was found in the active site cleft of *B. circulans* xylanase [27,41]. Substrate positional binding in XT6 [26] revealed that not only the –OH of Glu58 and –NH_2_ of Asn59 at the −2 site could bind with the –OH of xylose, but also that Asn204 at the +2 site could form a hydrogen bond with the –O of xylose. The homologous comparison showed that GH10-xyn has those corresponding sites, which are Asn86 at the −2 site and Asn214 at +2, and the Asn86 sidechain NH_2_ group is especially likely to be important (Figure 3). From the predicted 3D structure of GH10-xyn via homology modeling, it seemed that the N86 residue was on the surface of the catalytic cleft and very close to the predicted proton donor (E169) or nucleophile (E227). Therefore, N86 and N214 of GH10-xyn and Y115 of GH11-xyn were chosen for site-directed mutagenesis to further study their roles in substrate binding.

**Figure 7:**
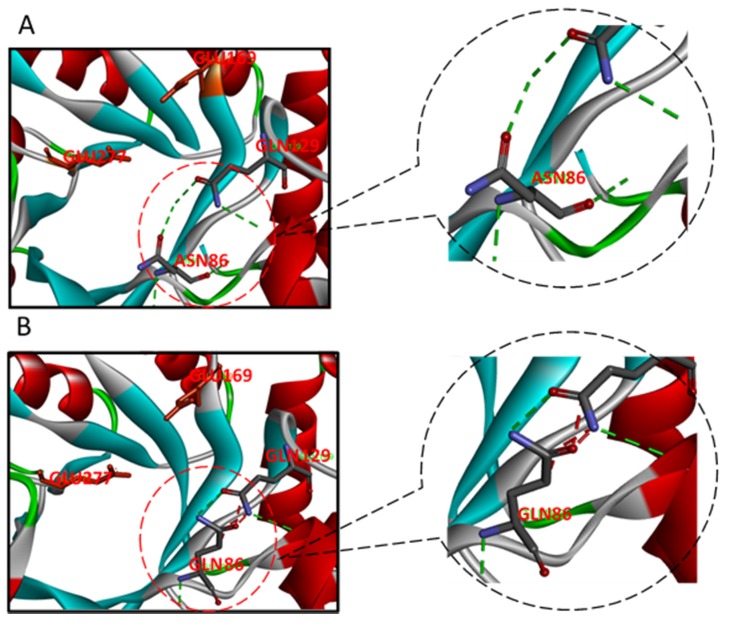
Modeling locations of the binding sites and catalytic sites of GH10-N86 (**A**) and GH10-N86Q (**B**). The green lines stands for favorable bump interaction, and the red lines stands for unfavorable bump interaction.

GH10-N86A and GH11-Y115A showed weakened −2 binding capability, because the asparagine NH2 group hydrogen bonding and the tryptophan hydrophobic stacking affect the structure of the catalytic domain, resulting in increased XOS proportion and production. For XOS with a higher DP, the mutants also showed a preference to attack the second xylosidic bond from the reducing end [42] in subsites −2 to +1, showing preferential binding for xylotriose. Thus, Ala substituted for Asn at the −2 subsite weakened substrate binding, while the Gln substitution retained the activity as predicted by the molecular structure modeling (Figure 7). In wild-type ±N subsites of GH10-xyn, Asn86 forms hydrogen bonds with Glu87, Lys89, and Gln129, which creates the\spatial configuration of the −2 location. Lys89, Asn86 and Gln129 show an impact on −2 with their interactions. GH10-N86Q has two amino groups from glutamine that form hydrogen bonds of Gln86: CD and Lys89: NZ that create unfavorable bump interactions, which could expand the pocket of the catalytic area. At the same time, the conformational change also pushes Q129 into the bonding disorder. While some hydrogen bonds still remain, such as Gln86: N, Glu87:OE1, Lys89: NZ, and Gln129:OE1, which could maintain catalytic activity, the N86A mutation canceled all glutamic acid bonds leading to a significate decrease of catalysis. Thus, among the mutants, GH10-N86Q had a consistent activity of GH10-xyn. However, its *K_cat_* decreased to 94.90 s^−1^ from 150.82 s^−1^ of the wild-type. We speculate that the Gln (GH10-N86Q) substituted for Asn at the −2 subsite retained the activity as predicted by the molecular structure modeling but GH10-N86Q site-directed mutagenesis at the −2 site partially weakened the affinity of the substrate and enzyme. Thus, GH10-N86Q had the consistent activity of GH10-xyn with decreasing *K_m_* value. Furthermore, the *K_cat_*/*K_m_* of GH10-N86Q also reflects decreasing affinity and catalytic ability of enzyme towards a substrate. This study demonstrated that the capacity of GH10 xylanases to hydrolyze and produce low DP XOS can be ascribed to the strong binding at subsites −2, −1, and +1 [43]. Especially, subsite −2 exhibits a high affinity for xylose residues [25,44], and mutation N44A at the −2 subsite causes a strong reduction in activity against XOS [25]. GH10-N214 did not produce a desired result, perhaps because losing capacity to bind substrate at the +2 subsite led to more binding on the +1 subsite, causing more X1 to appear.

### 3.3. Role of Substituent Group in Substrate of xynA in Hydrolysis

Oat-spelt xylan comes from a cereal grain and commonly contains arabino-xylan (AX) due to the large proportion of l-arabinose attached to the backbone [43]. Glucuronoxylans of hardwoods such as birch and beechwood are substituted with MeGlcA every 10–20 X1 residues that are α-1, 2 linked to the X1 residues of the backbone [45]. GH10 xylanases can attack the glycosidic linkage next to a single-or double-substituted xylose toward the non-reducing end and require two unsubstituted xylose residues between glucuronoxylan branched residues [16,28,46] and AX [23,47]. For each subsite, some studies have shown different possible conformations so that single a-L-arabinofuranosyl has more sites than MeGlcA [48]. Moreover, GH10-xyn shows higher activity against XOS and produces lower DP hydrolysis products from glucuronoxylan and AX than GH11 [16,47].

In conclusion, our results indicated that site-directed mutagenesis at the −2 binding site weakened the ability to hydrolyze X3 of GH10-xyn from *Streptomyces* L10608, which is important for increasing the utilization efficiency of hemicellulosic materials. Although the mechanism of the improvement is not yet well elucidated, this study provides a rationale for large-scale commercial XOS production by xylanases with altered binding.

## 4. Methods and Materials

### 4.1. Plasmids and Microorganisms

Xylose (X1), xylobiose (X2), xylotriose (X3), xylotetraose (X4) and xylopentaose (X5) were provided by Megazyme (Wicklow, Ireland). All other substrates including xylans from birchwood, beechwood, and oat-spelt were obtained from Sigma (Sigma-Aldrich Co., Saint Louis, MO, USA). All other chemicals used were of chromatographic grade.

*E. coli* DH5α was used as a host for gene cloning and plasmid propagation. The cloning vector pMD18-T (TaKaRa, Bio Inc., kusatsu, Japan) was used for cloning and sequencing of PCR fragments. Prokaryotic expression strain BL21 (DE3) (TIANGEN, Beijing, China) was used for heterologous expression of xylanases in *E. coli*. All oligonucleotides used in this research were synthesized by Beijing AuGCT biotechnology Co., Ltd., (Beijing, China).

### 4.2. PCR Amplification and Sequence Determination of the Isolates for Strain Identification

*Streptomyces* L10608 (Beijing Technology and Business University, Beijing, China) cultured in liquid Luria-Bertani (LB) culture medium on a rotary shaker (200 rpm) at 37 °C in 24 h for strain identification. The 16S rRNA oligonucleotide sequence analysis was used for identifying the species of the strain L10608. Genomic DNA Isolation: Cultured cells were collected by centrifugation at 10,000× *g* for 5 min. Cells were washed with distilled water and ground into a powder for DNA extraction by using a bacterial DNA general kit (OMEGA Bio-tek, Inc., Norcross, GA, USA). The extracted DNA samples were tested by electrophoresis in 1% agarose gel (0.25 g agarose, 25 mL 1 × TAE buffer (Tris base, Acetic acid, Ethylenediaminetetraacetic acid)) with ethidium bromide as the chromogenic reagent.

PCR amplification of 16S rRNA: PCR was used with the bacterial forward primer Bac27F (5′-AGAGTTTGGATCMTGGCTCAG-3′) and universal reverse primer Univ1492R (5′-CGGTTACCTTGTTACGACTT-3′). The following standard conditions were used for bacterial 16S rRNA. The gene amplification: initial denaturation at 95 °C for 5 min; 35 cycles of denaturation (30 s at 95 °C), annealing (30 s at 55 °C), and extension (30 s at 72 °C); and a final extension at 72 °C for 10 min. PCR products were tested by electrophoresis in 1% (*w*/*v*) agarose gel stained with ethidium bromide as the chromogenic reagent. Phylogenetic analysis was realized by alignment of consensus sequences of 16S rRNA collected in an international database (GenBank, https://www.ncbi.nlm.nih.gov/genbank/). The homologue resultants were then expressed as a percentage between the submitted sequence and the most relevant sequences from the database. Then, the phylogenetic tree of strain was constructed using MEGA software (Version 7.0, https://www.megasoftware.net/).

### 4.3. Cloning and Overexpression of the Xylanase Gene

Total DNA from *Streptomyces* L10608 was extracted using a Bacterial DNA Kit (OMEGA D3350-00; OMEGA Bio-tek, Inc., Norcross, GA, USA). Cloning of the two genes encoding *GH11* and *GH10* from *Streptomyces* L10608 was performed by genomic walking and nested-PCR. Primers (Table S1) were designed for the conserved domain sequence, gene walking, and two cycles of nested-PCR [49]. 729 bp and 1431 bp PCR fragments were amplified with primers with *Xho*I/*Nco*I sites using cDNA and genomic DNA as the template, respectively. Then the two fragments were cloned into pMD18-T and sequenced (AuGCT, Beijing, China).

Genes encoding mature *GH10* and *GH11* were cloned into the *Xho*I/*Nco*I sites of the pET-28a (+) vector (TIANGEN Bio-tek Co., Ltd., Beijing, China), which was then transformed into *E. coli* BL21 (DE3) strain to induce gene expression with Isopropyl β-d-Thiogalactoside (IPTG). Transformants were screened on Luria-Bertani (LB) agar plates containing kanamycin. The selected transformants were cultured in a flask containing LB medium at 37 °C and induction expressed by adding 1 mM IPTG until the optical density of the culture measured at 600 nm (OD600) was 0.6. After culturing for 12 h at 20 °C, the cells harvested by centrifugation and disrupted by ultrasonication. Then the recombinant xylanase was purified by nickel-affinity chromatography. The recombinant crude protein solution was purified by the Akta Fast protein liquid chromatography (FPLC) protein purification system. The buffer 50 mM NaH_2_PO_4_ (0.5 M NaCl, 0 mM imidazole, pH 8.0) to balance the column material, the unspecific binding protein was washed down using the buffer 50 mM NaH_2_PO_4_ (0.5 M NaCl, 5 mM imidazole, pH 8.0). Elution: the gradient of the recombinant protein was performed by the different concentration of NaH_2_PO_4_-imidazole buffer, the A280 value was detected, and the lotion was collected immediately after the peak. Collection enzyme at 4 °C for later research.

### 4.4. Site-Directed Mutagenesis of the Xylanase Genes

Site-directed mutagenesis and creation of GH10-ΔR were carried out by gene splicing by overlap extension PCR (SOEPCR) [31]. The primers (Table S2) were used for creating mutants Asn214 (+2), Asn86 (−2), and GH10-ΔRicin R (without the Ricin B-lectin domain) by Ala or Gln in GH10 xylanase, and Y115A (−2) and Y123A (−2) in GH11 xylanase. The resulting DNA fragments were also ligated into the same expression vector and induced as described above.

### 4.5. 3D Models of Recombinant Proteins

An existing 3D model of xylanase was used to analyze the potential binding sites of xylanase from the L10608 strain, which has highly conserved sequences in its catalytic domain (1HIX, the model template of GH11-xyn, 84.4% similarity; 1XYF, the model template of GH10-xyn, 72.6% similarity is GH11-xyn) [21,50]. The 3D structure of the xylanase was constructed by SWISS-MODEL software (https://www.swissmodel.expasy.org/) and analyzed by AutoDockTools (Version 1.5.6, http://autodock.scripps.edu/).

### 4.6. Xylanase Enzymatic Assays and Kinetics Parameters

To determine xylanase activity and enzyme characteristics, the wild-type and mutants were further purified using the Ni-sepharose, purified and suitably diluted xylanase 50 μL was added to 450 μL 10 g·L^−1^ of birchwood xylan substrate. The reactions were performed at series of pH and temperature for 5 min along with heat-inactivated xylanase as a control and were stopped by adding 0.5 mL 3,5-dinitrosalicylic acid (DNS) solution. The released reducing sugars were measured by the DNS method using X1 as a standard. One unit (U) of the xylanase activity was defined as an amount of the enzyme capable of releasing 1 μM of reducing sugar from xylan with concentration of 2 mg·mL^−1^ at 25 °C in pH 5.5, per 1 min. The effect of varied pH values on the activity of xylanase was studied at 55 °C in a range of pH 4.0–8.0 (20 mM): citrate buffer for pH 4.0–6.5; phosphate buffer for pH 7.0–8.0; and at 35 to 80 °C to explore the optimal temperature at pH 5.5. For the kinetic experiments, purified xylanase activity assays were performed with six different concentrations of substrates under the conditions of optimal pH 5.5, and optimal temperature 55 °C, then the *K_m_* and *K_cat_* values were calculated from the kinetic data using the GraphPad Prism software (Version 7.0, https://www.graphpad.com/).

### 4.7. Hydrolysis of XOS and Xylans by Purified Xylanases

0.1 mg of XOS (DP was 3 to 5) was separately mixed with 10 U of purified xylanases to study the hydrolysis mechanism, in pH 5.5 acetate-acetate buffer solution at 50 °C for 12 h. To determine the influence of mutations on the hydrolysis products, 1 mg each of birchwood xylan, beechwood xylan, and oat-spelt xylan were separately hydrolyzed by 10 U of wild-type and mutant xylanases. The reactions were at the same conditions and the products were analyzed by High Performance Liquid Chromatography (HPLC) as described below.

### 4.8. Analysis of the Hydrolysis Products

Xylooligomers (0.1 mg each) and birchwood, beechwood, and oat-spelt xylan (1 mg) were reacted using purified wild-type and mutant xylanases (10 U) in 1 mL of 50 mM sodium acetate-acetate buffer solution (pH 5.5) for 12 h at 50 °C. Then the hydrolytic reaction was stopped by heating the reaction mixture at 100 °C for 10 min. The degradation products were identified by high performance liquid chromatography (HPLC) analysis using an Agilent 1200 Series HPLC system (Agilent Co., Santa Clara, CA, USA) equipped with a Sugar-D column (4.6 Inner Diameter (ID) × 250 mm, COSMOSIL, Nacalai tesque, Kyoto, Japan). The mobile phase contained acetonitrile and 30% water at a flow rate of 0.8 mL·min^−1^, oven temperature 30 °C, refractive index detector temperature 30 °C, and injection volume of 20 μL after using a 0.22 μm filter.

### 4.9. Statistical Analyses

Analysis of variance (ANOVA) and Duncan′s multiple range tests (at *p* ≤ 0.05) were performed to analyse significant differences and discriminate between the means. The values are shown as the means of two different tests with triplicate treatments per experiment.

## Figures and Tables

**Table 1:** Xylanase activities determined on Michaelis constant (*K_m_*), turnover number (*K_cat_*) and specificity constants (*K_cat_*/*K_m_*) of GH10/11-xyn and mutants towards soluble beechwood xylan.

Xylanse	Xylanase Activity (%)	Modified Subsites (s)	Kinetic Parameter		
			*K_m_* (mg·mL^−1^)	*K_cat_* (s^−1^)	*K_cat_*/*K_m_* (mg^−1^·s^−1^·mL)
GH10-xyn	100.00 ^a,b^	/	1.64 ^e^	150.82 ^b^	91.81 ^a^
GH10-N86A	8.28 ^d^	−2	13.01 ^b^	10.68 ^e^	0.82 ^e^
GH10-N86Q	125.71 ^a^	−2	1.85 ^e^	94.90 ^c^	51.29 ^b^
GH10-ΔR	47.47 ^b^	/	1.63 ^e^	54.93 ^d^	33.60 ^c^
GH10-N214A	25.95 ^c^	+2	2.83 ^d^	49.68 ^d^	17.56 ^d^
GH11-xyn	100.00 ^a,b^	/	35.88 ^a^	541.67 ^a^	15.10 ^d^
GH11-Y115A	6.25 ^e^	−2	6.25 ^c^	0.62 ^f^	0.10 ^e^

Means with the same superscript letter are not significantly different (*p* < 0.05).

**Table 2:** Xylooligosaccharides composition in hydrolysis products with xylan substrates (DP = 3–5).

Substrate	Enzyme	Hydrolyzates (%)			
		X1	X2	X3	X4
X3	GH10-xyn	51.22 ^b^	48.78 ^b^	ND ^d^	ND ^a^
	GH10-N86A	3.23 ^e^	ND ^e^	96.77 ^a^	ND ^a^
	GH10-N86Q	0.22 ^f^	1.25 ^f^	98.53 ^a^	ND ^a^
	GH10-ΔR	28.20 ^c^	71.80 ^a^	ND ^d^	ND ^a^
	GH10-N214A	28.21 ^c^	71.79 ^a^	ND ^d^	ND ^a^
	GH11-xyn	58.76 ^a^	4.52 ^d^	36.72 ^c^	ND ^a^
	GH11-Y115A	13.81 ^d^	5.37 ^c^	80.82 ^b^	ND ^a^
X4	GH10-xyn	10.94 ^c^	89.06 ^a^	ND ^c^	ND ^d^
	GH10-N86A	ND ^d^	31.46 ^c^	10.11 ^a^	58.43 ^a^
	GH10-N86Q	1.72	27.24	61.8	9.24
	GH10-ΔR	10.39 ^c^	89.61 ^a^	ND ^c^	ND ^e^
	GH10-N214A	11.22 ^c^	80.33 ^b^	ND ^c^	8.45 ^c^
	GH11-xyn	60.80 ^a^	31.81 ^d^	7.39 ^b^	ND ^d^
	GH11-Y115A	53.63 ^b^	25.16 ^e^	ND ^c^	21.21 ^b^
X5	GH10-xyn	15.32 ^c^	65.05 ^c^	ND ^d^	19.63 ^b^
	GH10-N86A	ND ^e^	28.87 ^d^	52.92 ^a^	18.21 ^b^
	GH10-N86Q	2.18	25.54	56.83	15.45
	GH10-ΔR	9.09 ^d^	90.91 ^a^	ND ^d^	ND ^d^
	GH10-N214A	14.58 ^c^	85.42 ^b^	ND ^d^	ND ^d^
	GH11-xyn	58.96 ^a^	16.76 ^e^	16.19 ^b^	8.09 ^c^
	GH11-Y115A	37.5 ^b^	25.00 ^f^	8.33 ^c^	29.17 ^a^

ND, not detected. Means with the same superscript letter are not significantly different (*p* < 0.05).

**Table 3:** Xylooligosaccharides composition in hydrolysis products with different polymeric substrates.

Substrate	Enzyme	Hydrolyzates (%)			
		X1	X2	X3	X4
Beechwood xylan	GH10-xyn	20.66 ^b^	78.90 ^a^	ND ^c^	0.44 ^d^
	GH10-N86A	0.38 ^e^	4.07 ^e^	53.70 ^a^	41.85 ^a^
	GH10-N86Q	2.49 ^d^	27.78 ^d^	53.76 ^a^	15.97 ^b^
	GH10-ΔR	13.67 ^c^	84.85 ^a^	0.91 ^c^	0.57 ^d^
	GH10-N214A	40.61 ^a^	58.21 ^b^	0.71 ^c^	0.47 ^d^
	GH11-xyn	15.90 ^c^	42.42 ^c^	40.4 ^b^	1.28 ^c^
	GH11-Y115A	ND ^e^	25.77 ^d^	57.85 ^a^	16.38 ^b^
Oat spelt xylan	GH10-xyn	14.20 ^b^	77.9 ^a^	3.81 ^d^	4.09 ^d^
	GH10-N86A	ND ^e^	26.08 ^d^	64.83 ^a^	9.09 ^c^
	GH10-N86Q	1.45 ^e^	17.8 ^e^	46.53 ^c^	34.22 ^a^
	GH10-ΔR	23.27 ^a^	72.67 ^b^	2.03 ^e^	2.03 ^e^
	GH10-N214A	26.23 ^a^	70.00 ^b^	1.76 ^e^	2.01 ^e^
	GH11-xyn	10.08 ^c^	45.80 ^c^	43.07 ^c^	1.05 ^e^
	GH11-Y115A	4.00 ^d^	16.00 ^e^	52.00 ^b^	28.00 ^b^
Birchwood xylan	GH10-xyn	21.23 ^a^	77.40 ^b^	ND ^e^	1.37 ^e^
	GH10-N86A	ND ^d^	25.59 ^d^	64.76 ^a^	9.65 ^d^
	GH10-N86Q	1.01 ^c^	25.68 ^d^	51.89 ^b^	21.42 ^a^
	GH10-ΔR	13.91 ^b^	84.60 ^a^	1.06 ^d^	0.43 ^e^
	GH10-N214A	19.17 ^a^	80.43 ^a^	ND ^e^	0.40 ^e^
	GH11-xyn	11.71 ^b^	33.65 ^c^	42.04 ^c^	12.60 ^c^
	GH11-Y115A	3.19 ^c^	26.06 ^d^	53.2 ^b^	17.55 ^b^

ND, not detected. Means with the same superscript letter are not significantly different (*p* < 0.05).

## Supplementary Materials

The following are available online at http://www.mdpi.com/1422-0067/19/3/834/s1.
