# Supplementary material for: Improving Hydrolysis Characteristics of Xylanases by Site-Directed Mutagenesis in Binding-Site Subsites from Streptomyces L10608
Source: Int J Mol Sci. 2018 Mar 13;19(3):834. doi: 10.3390/ijms19030834 (PMC5877695; doi:10.3390/ijms19030834)
Supplement: Supplementary file 1 [file ijms-19-00834-s001.docx]

**Improving hydrolysis characteristics of xylanases by site-directed mutagenesis in binding-site subsites from *Streptomyces* L10608**

Ke Xiong^a,b^, Su-yue Xiong^a,c^, Si-yu Gao^a,d^, Qin Li^a,c^, Sun-bao Guo^a,b^, Xiu-ting Li^a,b*^

^a^Beijing Innovation Centre of Food Nutrition and Human, Beijing Technology & Business University (BTBU), No. 33 Fucheng Road, Haidian, Beijing 100048, China

^b^ Beijing Laboratory for Food Quality and Safety, Beijing Technology & Business University (BTBU), No. 33 Fucheng Road, Haidian, Beijing 100048, China

^c^ Beijing Engineering and Technology Research Center of Food Additives, Beijing Technology & Business University (BTBU), No. 33 Fucheng Road, Haidian, Beijing 100048, China.

^d^ Beijing Key Laboratory of Flavor Chemistry，Beijing Technology and Business University (BTBU), Beijing 100048, China.

**Running title:** Improving special hydrolysis characterization of strain by mutagenesis

*Corresponding author. Tel: +86-10-6898-4547; Fax: +86-10-6898-4547;

E-mail addresses: xiongke@btbu.edu.cn

**Supplementary data**

**Table S1** Gene walking primers used in study

| Primer | Sequence(from 5’ to 3’） |
| --- | --- |
| GH11-F | CGCCGAGAAGGGCCGCTACT |
| GH10-F | GCGKDRTASGCGGSCTTCTTG |
| GH11-R | AGTGCGACTGGAAGCCGAC |
| GH10-R | GTGACSSCCGAGAACGAGAT |
| LAD1-1 | ACGATGGACTCCAGAGCGGCCGCVNVNNNGGAA |
| LAD1-2 | ACGATGGACTCCAGAGCGGCCGCBNBNNNGGTT |
| LAD1-3 | ACGATGGACTCCAGAGCGGCCGCVVNVNNNCCAA |
| LAD1-4 | ACGATGGACTCCAGAGCGGCCGCBDNBNNNCGGT |
| AC1 | ACGATGGACTCCA GAG |
| GH-10xynWL-F1 | CCGTCCGTCAGAAGCTCCGAACC |
| GH-10xynWL-F2 | GCGTGATGAACCACTCAAAGGGCA |
| GH-10xynWL-R1 | AACGGGAGACGGCCAGGCAGT |
| GH-10xynWL-R2 | TTTCAGGTGCGGGTCCAGCGTT |
| GH-11xynWL-F1 | GATCATGTAGTACCGGAACTGGCCC |
| GH-11xynWL-F2 | ACGATGGACTCCAGTCCGGCCGCGTTGTAGCGGGTCGTCTTGT |
| GH-11xynWL-R1 | CACCATCACCACGGGGAATCAC |
| GH-11xynWL-R2 | ACGATGGACTCCAGTCCGGCCAAGGCTACCAAAGCAGCGGAAACA |
| GH-11NcoF | CATGCCATGGCAATGCACCAGGACGGTTCGCAGC |
| GH-11XhoR | CCGCTCGAGGCCGCTGACCGTGATCGTGGAG |
| GH-10NcoF | CATGCCATGGCAATGGGCATCCAAGCCCTTCCCA |
| GH-10XhoR | CCGCTCGAGGGTGCGGGTCCAGCGTTGGTT |

P.S.：R=A/G，S=C/G，W= A/T，B= C/G/T，N=A/C/G/T; Gene walking primers of LAD1-1, LAD1-2, LAD1-3 and LAD1-4 were design according to reference (Primers P.2007.High-efficiency thermal asymmetric interlaced PCR for amplification of unknown flanking sequences. Biotechniques 43: 649-656.)

**Table S2** Site -directed point mutation primers used in study

| Primer | Sequence(from 5’ to 3’） |
| --- | --- |
| GH10- xyn△R | CCGCTCGAG GTCACCGGTCGGCGGGGG |
| GH10-xyn N214A R | CCAGTTCTCGACGGCGTAGTCGTTG |
| GH10-xyn N214A F | CAACGACTACGCCGTCGAGAACTGG |
| GH10-xyn N86A R | TCTTCATCTCGGCCTCGGCGGT |
| GH10-xyn N86A F | ACCGCCGAGGCCGAGATGAAGA |
| GH10-xyn N86Q R | CTTCATCTCGCCCTCGGCGGT |
| GH10-xyn N86Q F | ACCGCCGAGGGCGAGATGAAG |
| GH11-xyn W69A R | GCCGTCGGTGGCGAACGAGTAGTAC |
| GH11-xyn W69A F | GTACTACTCGTTCGCCACCGACGGC |

**Figure S1** Nucleotide sequences of the genes

**GH11-xyn nucleotide sequences:**

1 ATGCACCAGG ACGGTTCGCA GCAGGACAGG ACCCAGAACC CCGCCCCCTT CGGCGGCCTG

61 AGCCGGCGAG GCTTCCTCGT CGGGGCCGGT ACGGGCGCCG CCGCGCTCGC CGCCGGGTCC

121 GGGCTGCTGC TGCCCGGCAC CGCACACGCC GCGACCACCA TCACCACCAA CCAGACCGGC

181 TACGACGGCA TGTACTACTC GTTCTGGACC GACGGCGGCG GCTCCGTGTC CATGACGCTC

241 AACGGAGGCG GCAGCTACAG CACCCGGTGG ACCAACTGCG GCAACTTCGT CGCCGGCAAG

301 GGCTGGAACA ACGGTGGACG CAGGACGGTC CGCTACACCG GCTACTTCAA CCCGTCGGGC

361 AACGGATACG GCTGCCTCTA CGGCTGGACC TCGAACCCGC TCGTCGAGTA CTACATCGTC

421 GACAACTGGG GCAGCTACCG CCCCACCGGC GAGTACCGGG GCACGGTCTA CAGCGACGGC

481 GGCACCTACG ACATCTACAA GACGACCCGC TACAACGCCC CGTCCGTCGA GGGCACCCGC

541 ACCTTCGACC AGTACTGGAG CGTCCGGCAG TCCAAGGTGA CCAGCGGCTC CGGCACCATC

601 ACCACGGGGA ATCACTTCGA CGCGTGGGCG CGTGCCGGTA TGAACCTGGG CCAGTTCCGG

661 TACTACATGA TCATGGCTAC CGAAGGCTAC CAAAGCAGCG GAAGCTCCAC GATCACGGTC

721 AGCGGCTGA

**GH10-xyn nucleotide sequences:**

1 ATGGGCATCC AAGCCCTTCC CAGAGCCGCC GTCCGTCAGA AGCTCCGAAC CCCGCTGCTG

61 GCGCTGGCCG CCGGCGTCCT CGGGCTGACG GCCGCACTCG TCCCGCCCAC GAACGCGGAC

121 GCCGCCGAGA GCACCCTCGG CGCCGCCGCG GCGCAGAGCG GCCGCTACTT CGGCGTCGCC

181 ATCGCCTCGG GCAAGCTCGG CGACTCGACG TACACCTCGA TCGCAAACCG CGAGTTCAAC

241 TCGGTGACCG CCGAGAACGA GATGAAGATC GACGCCACCG AGCCGAACCG GGGCCAGTTC

301 AACTTCAGTT CGGCCGACCG CGTCTACAAC TGGGCCGTGC AGAACGGCAA GGAGGTCCGC

361 GGCCACACCC TCGCCTGGCA CTCCCAGCAG CCCGGCTGGA TGCAGAGCCT CAGCGGCAGC

421 TCGCTGCGCC AGGCCATGAT CGACCACATC AACGGCGTGA TGAACCACTA CAAGGGCAAG

481 ATCGCCCAGT GGGACGTCGT GAACGAGGCC TTCGCCGACG GCAGTTCGGG CGCCCGCCGC

541 GACTCCAACC TCCAGCGCAC CGGCAACGAC TGGATCGAGG TCGCCTTCCG CACCGCGCGC

601 GCCGCCGACC CGTCCGCCAA GCTCTGCTAC AACGACTACA ACGTCGAGAA CTGGAACTGG

661 GCGAAGACCC AGGCCATGTA CAACATGGTC AAGGACTTCA AGTCGCGCGG CGTGCCGATC

721 GACTGCGTCG GCTTCCAGTC GCACTTCAAC AGCGGCAGCC CGTACGACAG CAACTTCCGC

781 ACCACCCTGC AGAACTTCGC GGCCCTCGGC GTCGACGTCG CCGTCACCGA GCTCGACATC

841 CAGGGCGCCT CGTCCTCGAC GTACGCCGCC GTGGTCAACG ACTGCCTGGC CGTCTCGCGC

901 TGCCTCGGCG TGACCGTCTG GGGTGTGCGC GACAGCGACT CCTGGCGTGC CAGTGACACG

961 CCGCTGCTGT TCAACAACGA CGGCAGCAAG AAGGCCGCGT ACTCCGCCGT CCTCAACGCG

1021 CTCAACGGCG GCACCACCAC GCCCCCGCCG ACCGGTGACG GCGGTCAGAT CAAGGGCGTC

1081 GCCTCGGGCC GCTGCCTGGA CGTCCCCAAC GCCTCCACCA CCGACGGCAC AGGCGTCCAG

1141 CTGTACGACT GCCACTCCAA CAGCAACCAG CAGTGGGCCG TGACCGACTC CGGTGAGATC

1201 CGGGTCTACG GCAACAAGTG CCTGGACGCC GCCGGCACCG GCAACGGCGC CTCGGTCCAG

1261 ATCTACAGCT GCTGGGGCGG CGACAACCAG AAGTGGCGGC TGAACTCCGA CGGTTCGATC

1321 GTCGGCGTCC AGTCGGGCCG CTGCCTGGAC GCGGCCGGCA GCGGCAACGG CGCCAGGATC

1381 CAGCTCTACG CCTGCTCGGG CGGCTCCAAC CAACGCTGGA CCCGCACC
